# Interleukin-6 induces spatially dependent whole-body hypersensitivity in rats: implications for extracephalic hypersensitivity in migraine

**DOI:** 10.1186/s10194-021-01286-8

**Published:** 2021-07-13

**Authors:** Amanda Avona, Theodore J Price, Gregory Dussor

**Affiliations:** School of Behavioral and Brain Sciences, Center for Advanced Pain Studies, University of Texas at Dallas, Richardson, TX 75080 USA

**Keywords:** Migraine, Cephalic hypersensitivity, Interleukin-6, Headache, Referred hypersensitivity, Allodynia, Meninges, Dura mater, Central sensitization

## Abstract

**Background:**

Migraine is a complex neurological disorder that is characterized by throbbing head pain, increased sensitivity to light, sound, and touch, as well as nausea and fatigue. It is one of the most common and most disabling disorders globally but mechanisms causing migraine are poorly understood. While head pain is a typical feature of attacks, they also often present with cutaneous hypersensitivity in the rest of the body. In contrast, primary pain conditions in the lower parts of the body are less commonly associated with cephalic hypersensitivity. Previous studies indicate that application of stimuli to the meninges of rodents causes cutaneous facial as well as hindpaw hypersensitivity. In the present study, we asked whether widespread hypersensitivity is a unique feature of dural stimulation or whether body-wide responses occur similarly when the same stimulus is given in other locations.

**Methods:**

Rats were given the same dose of IL-6 either via dural, intraplantar, subcutaneous, intramuscular, intracisternal, or intrathecal injection. Cutaneous facial and hindpaw allodynia was assessed using Von Frey following injection into each location.

**Results:**

Hindpaw allodynia was observed following dural and intraplantar injection of IL-6 in both males and females. Hindpaw allodynia was only observed in females following intracisternal and intrathecal IL-6 injections. In contrast, facial allodynia was only observed in either sex following dural and intracisternal injections, which would activate meningeal afferents and the trigeminal nucleus caudalis (TNC), respectively.

**Conclusions:**

Here we show that while stimulation of upper body regions with IL-6 including the meninges and brainstem can cause widespread hypersensitivity spreading to the paws, similar stimulation of the lower body does not cause the spread of hypersensitivity into the head. These data are consistent with the observations that whole body hypersensitivity is specific to conditions such as migraine where pain is present in the head and they may provide insight into co-morbid pain states associated with migraine.

## Background

Migraine is among the top 6 most common disorders globally in both men and women [1] and is the 2nd-most disabling disease world-wide [2]. Despite the prevalence of migraine, little is known about the pathophysiology. Migraine is characterized by throbbing head pain, cutaneous allodynia, nausea, and sensitivity to light and sounds. These symptoms vary among migraine patients, as do the triggers that spawn these attacks. Common self-reported triggers include stress, hormonal changes, changes in sleep-wake patterns, skipping meals, and consumption of alcohol and certain foods [3].

Migraineurs often report increased sensory sensitivity and cutaneous allodynia in extracephalic regions during migraine attacks [4–7]. This is in contrast to other pain conditions in the lower body which are not typically associated with hypersensitivity in the head. This suggests that migraine, and the concomitant activation of meningeal afferents that causes the headache phase, may have a distinctive circuitry that leads to widespread hypersensitivity. Numerous prior studies using rodent migraine models have shown a remarkably consistent finding that stimulation of the dura mater causes cutaneous hypersensitivity of both the facial skin as well as that of the hindpaw [8–12]. These prior studies thus show that headache-inducing conditions lead to widespread hypersensitivity in rodents as in humans. While it is not generally reported that facial hypersensitivity exists in models of pain in the lower spinal system, these studies typically do not test for the presence of cephalic responses. The purpose of the present work was to test, using the same stimulus applied to multiple locations throughout the rat, whether dural stimulation is unique in its ability to cause body-wide hypersensitivity.

While migraine pathology remains poorly understood, inflammation is thought to be involved. Debate exists about the type, sites, and role of inflammation in migraine [13], but it is thought that peripheral inflammatory stimuli cause activation and hyper-excitability of meningeal afferents. These signals are received by the trigeminal ganglia, which sends these signals to higher order neurons in the trigeminal nucleus caudalis (TNC) [14]. From the TNC, these signals are then processed by the cortex for the perception of head pain [15]. One particular inflammatory mediator that has been implicated in migraine is interleukin-6 (IL-6). IL-6 is a pro-inflammatory cytokine that is upregulated in the serum and blood of migraine patients during an attack [16–18]. We have shown previously that application of IL-6 on to the dura of female and male rats as well as mice results in cutaneous facial allodynia [8, 19, 20]. Importantly, these studies also showed that IL-6 applied to the rodent dura causes facial and hindpaw allodynia [8, 11]. Additionally, this same dose of IL-6 given onto the dura is capable of sensitizing male and female rats to respond to subthreshold doses of migraine relevant triggers such as lowered dural pH and dural calcitonin gene-related peptide (CGRP) [8, 19]. Furthermore, we have shown that IL-6 applied to the dura of male rats causes facial and hindpaw allodynia [8, 11]. Based on the link between IL-6 and migraine and headache-relevant behavioral responses in preclinical models with dural IL-6, we chose this stimulus as the probe to test whether activation of meningeal afferent neurons can cause differential spread of hypersensitivity compared to the same stimulus given elsewhere.

## Methods

### Animals

In this study, 12–14 week-old approx. 260-300 g female and approx. 300-350 g male Sprague-Dawley rats (Taconic; Rensselaer, NY) were used for all experiments. Animals were housed on a 12-h light/dark cycle with access to food and water ad libitum. Animals were housed in the facility for at least 72 h prior to handling and habituation of animals to testing rooms. All procedures were conducted with prior approval of the Institutional Animal Care and Use Committee at the University of Texas at Dallas.

### Rat cannula implantation and drug delivery

Dural injections in rats were administered via cannula at a total of 10 μl injections. Cannulation surgeries were performed according to previously published methods [19, 21, 22] in which animals were anesthetized initially at 5% isoflurane via a nose cone; once animals no longer demonstrated a paw pinch reflex, isoflurane was lowered to 2.5–3.5% for the entirety of the surgery. The scalp was incised longitudinally and retracted from the midline to expose the skull. Using a pin vise (Grainger Industries) set to a length of 1 mm, a 1 mm burr hole was created using a stereotaxic frame at the target coordinates to sit above the middle meningeal artery (8 mm AP, − 2 mm ML, 1 mm DV) to puncture the skull while leaving the dura intact. A guide cannula (Plastics One C313G/SPC gauge 22) was implanted into the burr hole using a stereotaxic frame and sealed using Vetbond (Vetbond). Two screws were inserted above the guide cannula on both sides of midline below bregma. Perm reline repair resin (Coltene, Altstätten, Switzerland) was used to anchor the cannula to the screws and skull. To prevent clogging, a dummy cannula (Plastics One 313 DC-SPC 0.014–0.36 mm fit 1 mm) was inserted into the guide cannula. Post-surgery, animals were given subcutaneous 8 mg/kg gentamicin diluted in sterile saline and 0.25 mg meloxicam self-administered via Mouse MD’s™ (MD275–0125) bacon flavored tablets, to prevent infection and for pain management, respectively. Animals were returned to their home cage and allowed to recover for 7 days.

### Intraplantar injections

Intraplantar injections were administered according to previously published methods [19]. Briefly, rats were anesthetized initially at 5% isoflurane via a nose cone, once animals no longer exhibited a pinch reflex, isoflurane was lowered to 2.5–3%, during which time animals were injected. These animals received a volume of 50 μl into the left hindpaw via injection with a 30-gauge 0.5 in. needle attached to a Hamilton syringe. Animals were kept under isoflurane for < 2 min. Following intraplantar injection the left paw was subject to von Frey.

#### Subcutaneous injections

Subcutaneous scalp injections were performed while animals were anesthetized under a nose cone under 2.5–3% isoflurane. Injections were administered where a dural cannula would otherwise be implanted in a volume of 10 μl via a 30-gauge 0.5 in. needle. In all cases animals were kept under anesthesia for less than 2 min.

#### Gastrocnemius injections

Injections into the gastrocnemius muscle were performed in a volume of 10 μl administered into the left gastrocnemius muscle while the animals were anesthetized with 2.5–3% isoflurane administered via a nosecone. Subsequently the left hindpaw was tested to see if any hypersensitivity had developed as a result of injection.

### Intracisternal injections

Intracisternal injections were administered in a volume of 10 μl at a rate of 1 μl/sec and performed as previously described [8, 19, 23]. A 25-gauge 1.5 in. needle was contorted approximately 7 mm from the tip at a 45° angle with the bevel facing outwards. The needle was attached to a 27-gauge Hamilton syringe. Animals were anesthetized for < 2 min under 2.5–3% isoflurane via a nose cone. The head of the animal was tilted forward at approximately a 120° angle to allow access to the cisterna magna. The needle was positioned above C1 and inserted through the cisterna magna along the midline.

### Intrathecal injections

Intrathecal injections were performed according to previously published methods [24]. Injections of either 10 μl IL-6 or vehicle were administered into the L5-L6 intervertebral space while animals were under 2.5–3% isoflurane administered via a nose cone. Injections were administered using a 30-gauge 0.5 in. needle.

### Von Frey testing

Rats were allowed to acclimate to testing room, chambers, and light conditions for 2 h a day for 3 days prior to facial testing. Rats were handled for a single 5-min session at 24-h prior to habituation to the behavior chambers. Only rats that met a baseline of 8 g facial withdrawal threshold and 15 g hindpaw withdrawal threshold were included in the study. Following establishment of baseline, animals were given their respective injections. Facial withdrawal thresholds were determined by applying von Frey filaments to the periorbital region of the face (the midline of the forehead at the level of the eyes) in an ascending/descending manner starting from the 1 g filament. Briefly, if an animal did not respond, increasing filament forces were applied until the 8 g filament was reached or until a response was observed. If the animal responded to a specific filament, decreasing filament forces were applied until the 0.4 g filament was reached or until there were no responses observed. If no responses are observed, i.e. facial baseline is reached, the filaments tested are 1 g, 2 g, 4 g, 6 g, 8 g. Hindpaw withdrawal thresholds were determined via the same paradigm with a maximum of beginning with the 2 g filament and with a maximum of 15 g and a minimum of 0.6 g if an animal reaches a baseline withdrawal threshold on the paw the filaments tested are 2 g, 4 g, 6 g, 8 g, 15 g. Following central administration of IL-6 both the left and right hindpaws were tested; the paw showing greater hypersensitivity was then tested to be included in the data set.

### Drugs

Rat recombinant IL-6 (R&D systems, cat: 506-RL-050/CF) was diluted to a concentration of 0.1 ng for all experiments. For dural, hindpaw, and gastrocnemius muscle injections IL-6 was diluted in synthetic interstitial fluid (SIF) comprised of 135 mM NaCl, 5 mM KCl, 10 mM HEPES, 2mMCaCl2, 10 mM glucose, and 1 mM MgCl2 (pH 7.4, 310 mOsm). For intracisternal injections IL-6 was diluted in artificial cerebrospinal fluid (aCSF) which was comprised of 125 mM NaCl, 26 mM NaHCO_3_, 1.25 mM NaH_2_PO_4_, 2.5 mM KCl, 1 mM MgCL_2_, 2 mM CaCl_2_, and 10 mM D-glucose (pH 7.4). For intrathecal injection IL-6 was diluted in 0.9% saline. Diluents were used as respective vehicles for all experiments.

### Experimental design and statistical analysis

In all experiments, investigators were blinded to which animals received drug in all experiments. Allocation of animals to treatment groups was randomized via the “blinder” who chose animal identification numbers from a bag of pre-labeled paper slips. Data here are presented as mean ± SEM. Data were analyzed at each time point via two-way ANOVA and followed by Bonferroni post-hoc assessment where appropriate. Prism (GraphPad) was used for all data analyses. Significance was set to *p* < 0.05 for all analyses.

## Results

### Dural IL-6 causes facial and hindpaw sensitivity in female rats

**Fig. 1:**
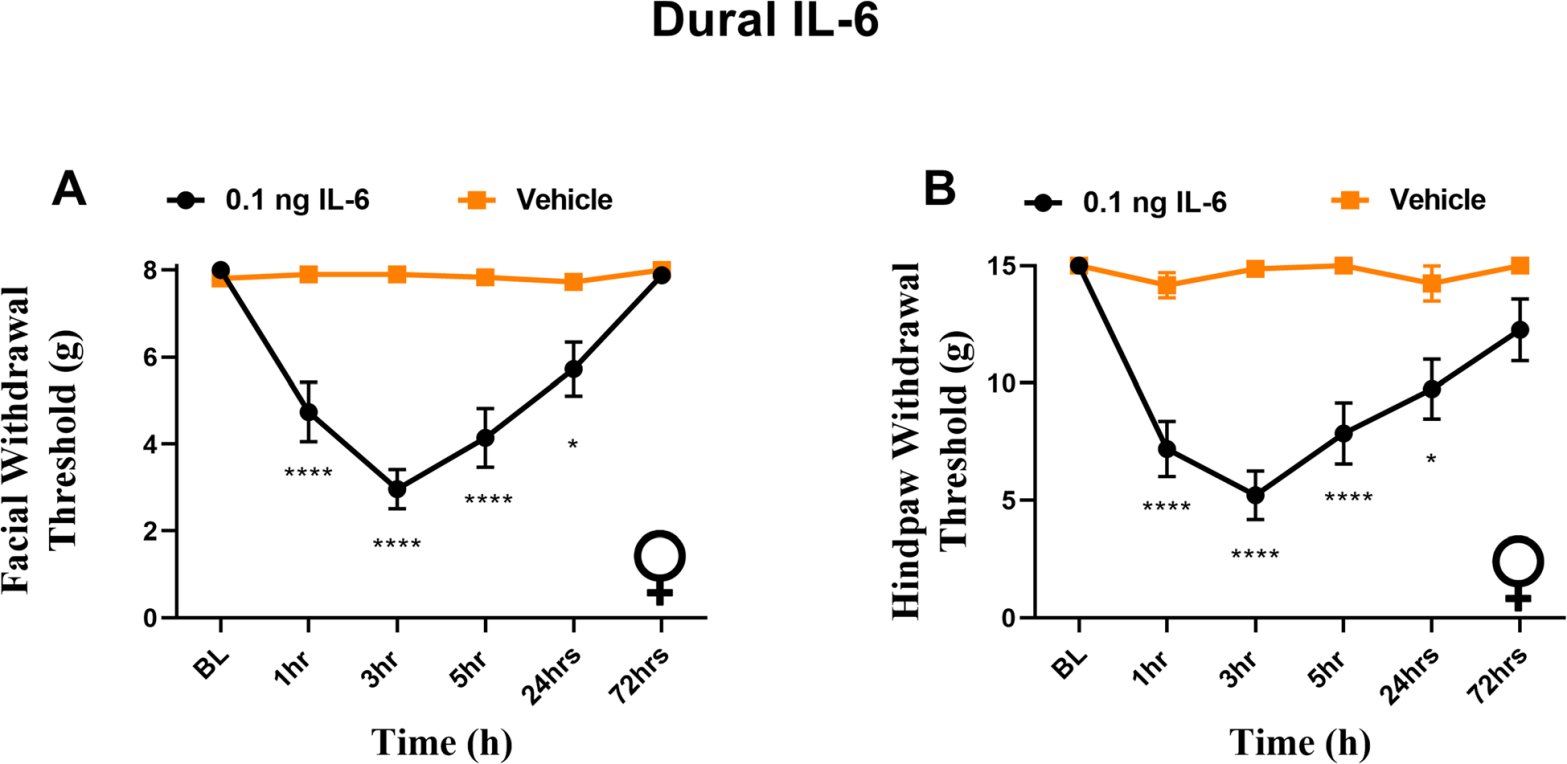
Dural IL-6 produces facial and hindpaw hypersensitivity in females. Female rats had baseline withdrawal thresholds established prior to receiving administration of 0.1 ng IL-6 onto the dura. Dural IL-6 (*n* = 10) elicited significant effect of treatment on facial (**A**) F (1, 66) = 31.58, *p* < 0.0001) and hindpaw (**B**) (F (1, 66) = 43.66, *p* < 0.0001) hypersensitivity when compared with animals that received vehicle (*n* = 4). *p < 0.05, ****p < 0.0001

Previously we found that application of IL-6 onto the dura of male rats not only resulted in facial hypersensitivity, but hindpaw sensitivity [8]. While we have additionally reported that dural IL-6 leads to facial allodynia in females [8], we did not test the ability of this stimulus to cause whole body allodynia in females. Given that cutaneous allodynia is more common in female migraineurs [25, 26] we aimed to test whether female rats would experience hindpaw allodynia similarly to male rats. Here we confirm that female rats that receive dural IL-6 demonstrate facial allodynia out to 72 h (Fig. 1A), and additionally experience significant hindpaw sensitivity, persisting for 24 h following injection (Fig. 1B).

### Intraplantar administration of IL-6 results in hindpaw, but not facial, allodynia

**Fig. 2:**
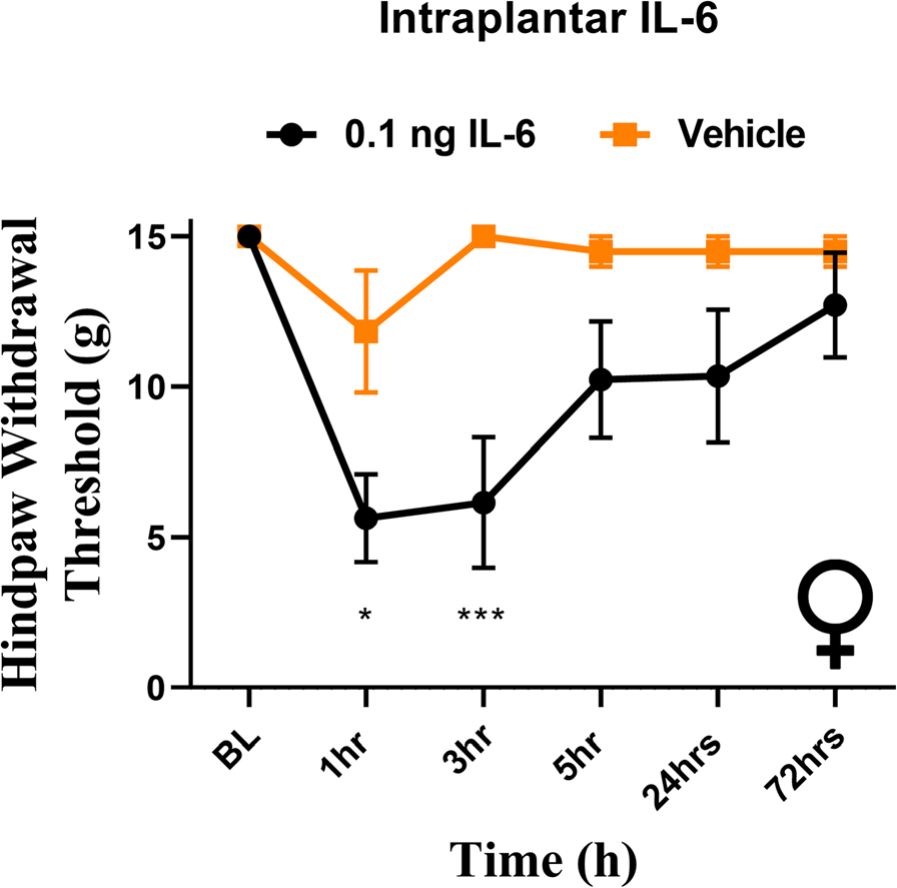
Intraplantar IL-6 produces hindpaw hypersensitivity in females. Female rats from the cohort also shown in Fig. 3 had baseline hindpaw withdrawal thresholds established prior to receiving intraplantar injection of 0.1 ng IL-6. IL-6 (*n* = 6) elicited significant effect of treatment on hindpaw hypersensitivity (F (5, 60) = 2.512, *p* = 0.0394) when compared with animals that received vehicle (*n* = 6). *p < 0.05, ***p < 0.001

**Fig. 3:**
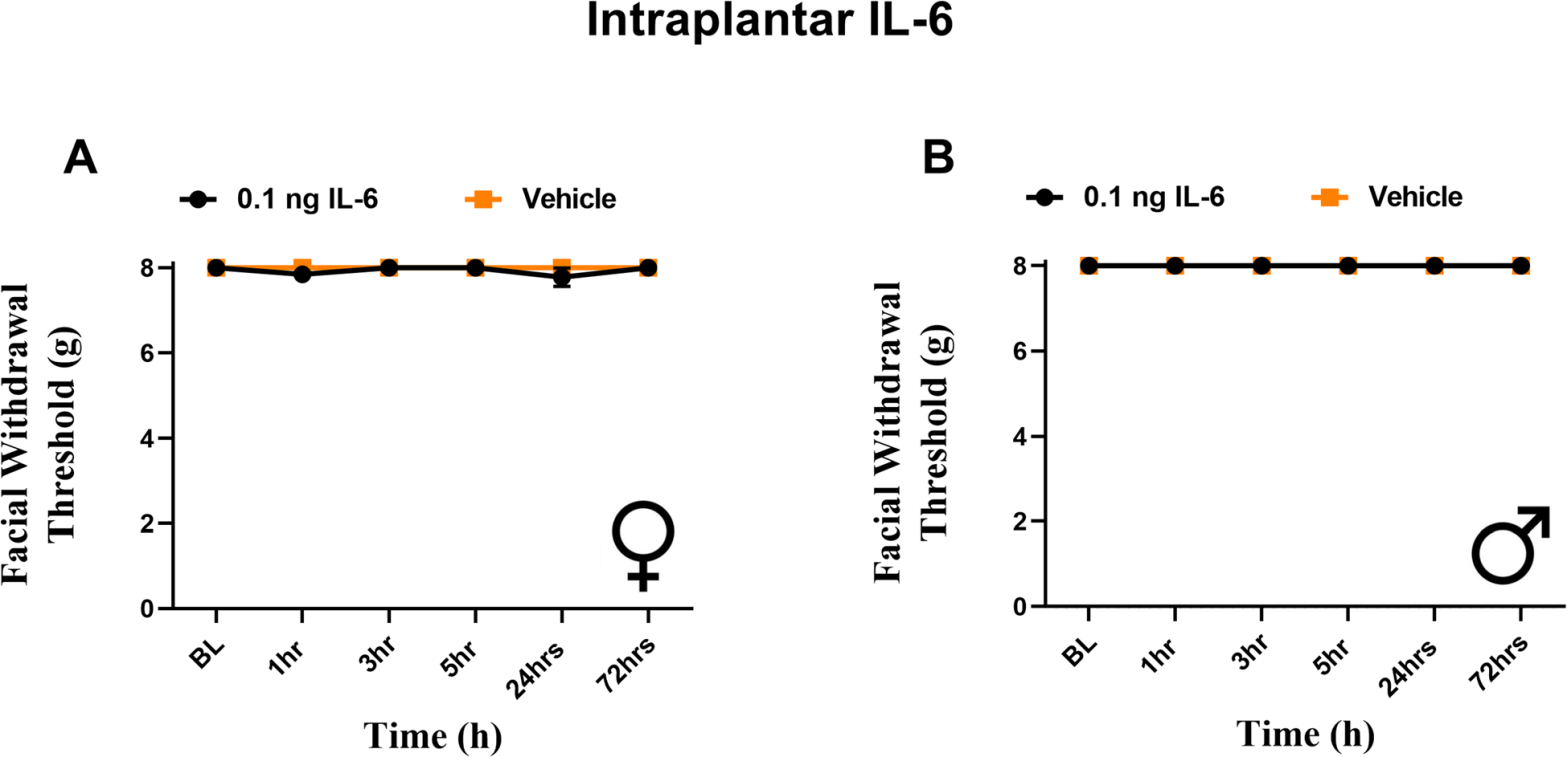
Intraplantar administration of IL-6 produces no facial responses in female or male rats. Female and male rats had facial hindpaw withdrawal thresholds assessed prior to and following intraplantar injection of 0.1 ng IL-6 (6 females, 6 males) or vehicle (6 females, 6 males). Two-way ANOVA followed by Bonferroni posthoc analysis revealed no significant facial responses in female (**A**) (F (1, 40) = 1.844, *p* = 0.1821) or male (**B**) (no variation among groups) rats

Our data here and in our prior publications indicates that dural stimulation with IL-6 causes referred hypersensitivity to the hindpaw. Previously we have reported that intraplantar IL-6 leads to sensitivity in the hindpaw [8]. We next sought to test whether intraplantar IL-6 causes periorbital hypersensitivity like that observed following dural IL-6. Both sexes were tested since females and males exhibit differential responses to migraine-relevant peptides in multiple peripheral tissues including the hindpaw [19, 27]. Both female and male rats received 0.1 ng IL-6 into the left hindpaw and were subject to periorbital and hindpaw von Frey testing. Females demonstrated acute hindpaw allodynia at 1- and 3-h following injection (Fig. 2), consistent with hindpaw responses shown previously with intraplantar IL-6 in males [8]. In contrast, neither females nor males exhibited any facial hypersensitivity responses at any time point following intraplantar IL-6 (Fig. 3). These data demonstrate that while dural IL-6 causes referred hypersensitivity to the hindpaw, intraplantar IL-6 does not cause referred hypersensitivity to the facial skin.

### Subcutaneous IL-6 in the scalp does not elicit facial or hindpaw hypersensitivity

**Fig. 4:**
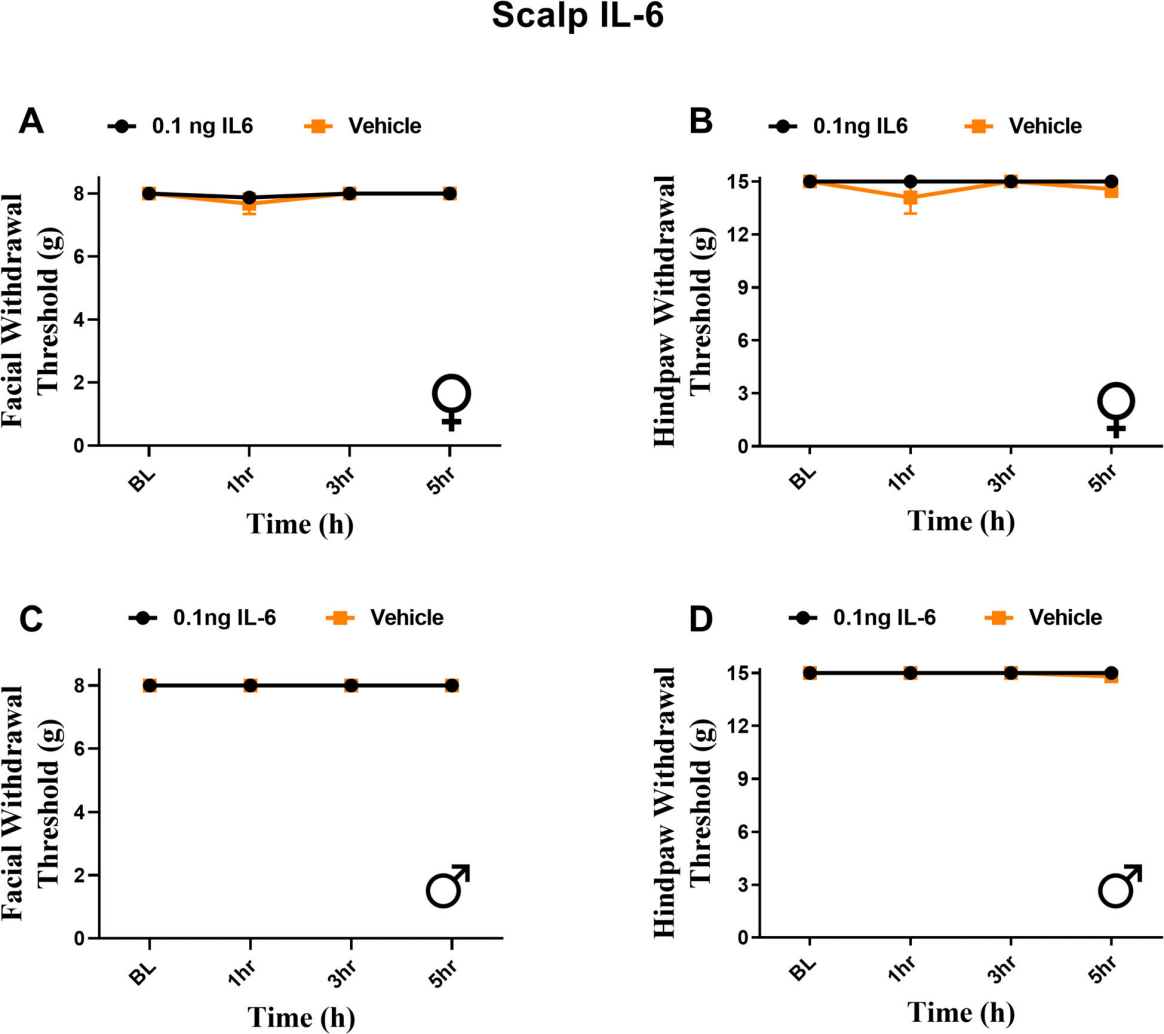
Subcutaneous injection of IL-6 in the scalp produces no significant facial or hindpaw responses in male and female rats. Rats had facial and hindpaw withdrawal thresholds assessed to establish baseline withdrawal thresholds, as well as following administration of 0.1 ng IL-6 (6 females, 8 males) or vehicle (7 females, 6 males) subcutaneously in the scalp. Two-way ANOVA followed by Bonferroni posthoc analysis revealed no significant facial or hindpaw responses in female (**A**, **B**) (F (1, 44) = 0.2761, *p* = 0.6019) (F (1, 44) = 1.502, *p* = 0.2269) or male (**C**,**D**) (no variation among groups) (F (1, 48) = 1.371, *p* = 0.2473) rats

Prior studies have found that migraine-relevant peptides injected into the periorbital region of the face of rats results in facial allodynia [28]. This is likely due to activation of fibers from the trigeminal ganglia that innervate the peri-orbital region of the face i.e. direct activation and sensitization of nerve endings near the site of von Frey testing. In contrast, for dural stimulation to cause periorbital hypersensitivity, central sensitization leading to referred responses from the dura to the facial skin must be present. This led us to question whether the periorbital hypersensitivity that results from 0.1 ng dural IL-6 would be observed if this stimulus was applied subcutaneously to the scalp where the dural cannula would otherwise be implanted. Thus, we administered subcutaneous IL-6 to the rostral part of the scalp in rats that were otherwise naïve, i.e., they had no dural cannula. Despite the noted projection of dural afferents to the extracranial periosteum [29], neither females nor males exhibited any periorbital (Fig. 4A, C) or hindpaw (Fig. 4B, D) hypersensitivity at any time point following subcutaneous IL-6. These data show that the referred periorbital hypersensitivity that develops following activation of dural afferents is not a general response to IL-6 injected anywhere in the head.

### Injection of IL-6 into the gastrocnemius muscle fails to produce facial or hindpaw hypersensitivity in male or female rats

**Fig. 5:**
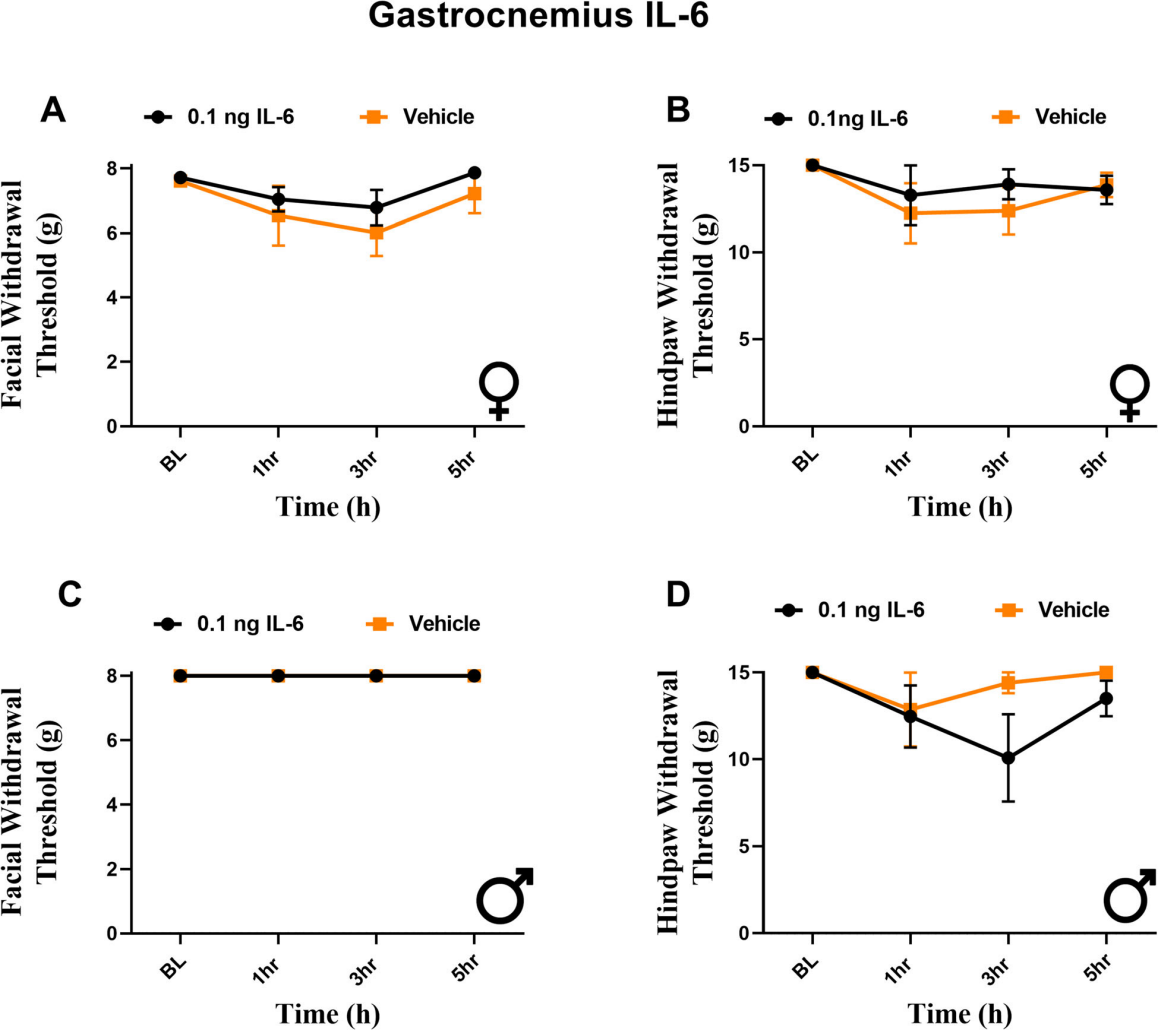
Gastrocnemius injection of IL-6 produces no significant facial or hindpaw responses in male and female rats. Rats had facial and hindpaw withdrawal thresholds assessed prior to and following administration of 0.1 ng IL-6 (6 females, 6 males) or vehicle (6 females, 5 males) into the gastrocnemius muscle. Two-way ANOVA followed by Bonferroni posthoc analysis revealed no significant effect of treatment on facial or hindpaw responses in female (**A**, **B**) (F (1, 40) = 1.844, p = 0.1821) (F (3, 36) = 0.2904, *p* = 0.8320) or male (**C**, **D**) (no variation among groups) (F (1, 36) = 2.289, *p* = 0.1390) rats

Peripheral cutaneous injections into the scalp produced no facial or hindpaw hypersensitivity, suggesting that whole body responses are not simply caused by general activation of the afferents within trigeminal system. One alternate possibility is that widespread referred responses are caused by activation of deep-tissue afferents but not cutaneous afferents. This is a well-known phenomenon observed with gastrointestinal or cardiac pain that is commonly referred to the surface of the abdomen. It is possible that hindpaw and scalp injections of IL-6 do not cause widespread referred hypersensitivity since they are subcutaneous while stimulation of the dura mater is more similar to activation of deep-tissue afferents. To address this possibility, we administered 0.1 ng IL-6 into the gastrocnemius muscle. We observed no significant reductions in facial or hindpaw withdrawal response to this intramuscular injection in either female (Fig. 5A, B) or male rats (Fig. 5C, D). This indicates that the referred hypersensitivity of the facial and hindpaw skin following dural stimulation with IL-6 is not a generalized response to activation of non-cutaneous afferents.

### Intracisternal IL-6 produces facial and hindpaw hypersensitivity in rats

**Fig. 6:**
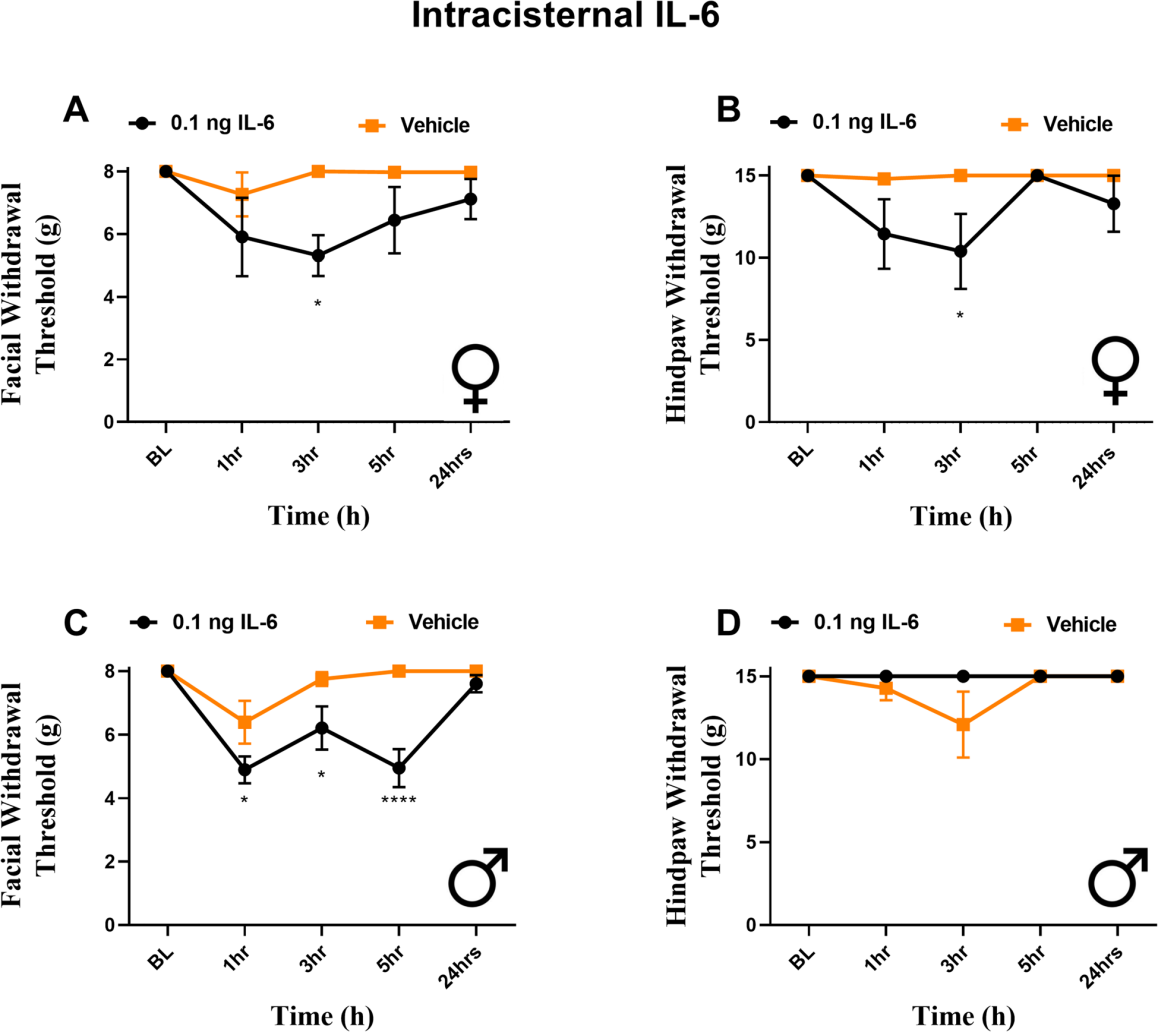
Intracisternal IL-6 produces facial hypersensitivity in both sexes, but differential hindpaw responses. Female (**A**, **B**) and male (**C**, **D**) rats had baseline withdrawal thresholds of the face and hindpaw determined prior to intracisternal injection of IL-6 (4 females, 6 males) or vehicle (5 females, 7 males). Both females and males demonstrated significant effects of treatment on facial responses (F (1, 35) = 12.24, *p* = 0.0013) (F (1, 55) = 27.98, *p* < .0001); however, only females presented with hindpaw responses. (F (1, 35) = 9.640, *p* = 0.0038) *p < 0.05, ****p < 0.0001

It has been shown previously that dural application of inflammatory soup (IS) can lead to sensitization of neurons within the TNC [14, 30, 31]. This sensitization that develops has been suggested to be the underlying mechanism of referred facial hypersensitivity following dural stimulation. We thus aimed to determine whether direct stimulation of the central terminals of trigeminal afferents or of second-order neurons within the TNC with IL-6 would produce whole body hypersensitivity. To test this, rats received 0.1 ng IL-6 into the cisterna magna. Female rats exhibited facial (Fig. 6A) and hindpaw (Fig. 6B) hypersensitivity at 3 h following injection. Male rats exhibited no hindpaw hypersensitivity (Fig. 6D) but demonstrated significant facial allodynia from 1 to 5 h following injection (Fig. 6C). These data show that activation of circuits within the brainstem by IL-6 is capable of causing referred facial hypersensitivity, and also referred paw hypersensitivity in females, but that only dural stimulation causes referred paw hypersensitivity in males.

### Intrathecal IL-6 causes hindpaw hypersensitivity in females

**Fig. 7:**
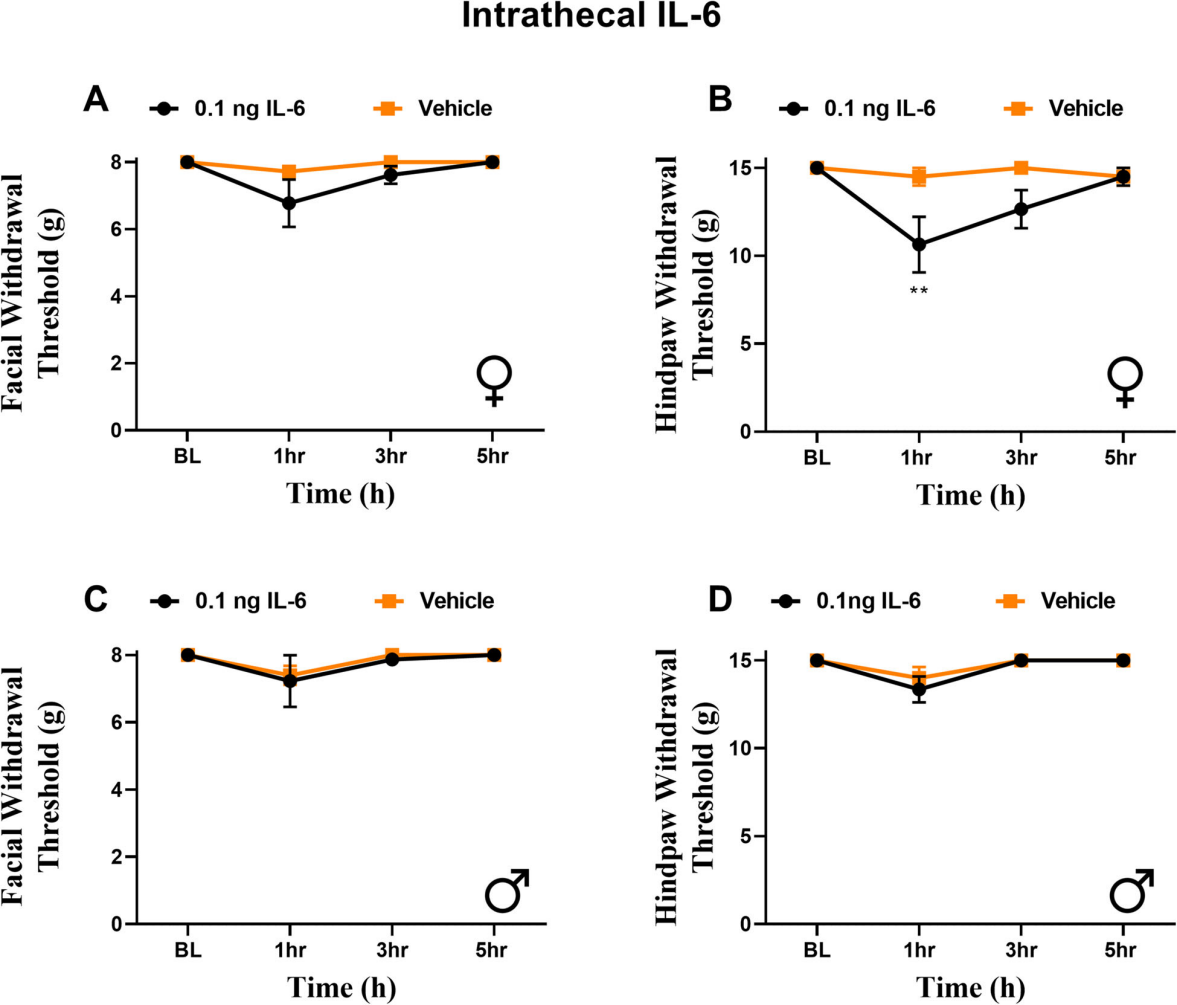
Intrathecal IL-6 produces hindpaw responses in female, but not male rats. Rats had periorbital and hindpaw withdrawal thresholds assessed prior to and following intrathecal administration of 0.1 ng IL-6 (6 females, 6 males) or vehicle (6 females, 6 males). Two-way ANOVA followed by Bonferroni posthoc analysis revealed no significant effect of treatment on facial responses in female (**A**, **B**) (F (1, 40) = 2.924, *p* = 0.0950) or male (**C**, **D**) rats (F (1, 40) = 1.280, *p* = 0.7223). Female rats demonstrated significant hindpaw allodynia. (F (1, 40) = 8.700, *p* = 0.0053) **p < 0.01

The findings showing that intracisternal injection of IL-6 leads to whole body hypersensitivity, at least in females, led us to question whether central sensitization in general leads to widespread hypersensitivity, or whether this is specific to the activation of the trigeminal pathways. To address this question, we administered 0.1 ng IL-6 intrathecally in both male and female rats. IL-6 produced significant acute hindpaw hypersensitivity in females 1 h following injection (Fig. 7B). No hindpaw hypersensitivity was observed in males at any time point (Fig. 7D). Importantly, no facial hypersensitivity responses were observed in either sex at any time point (Fig. 7A, C). These findings show that referred hypersensitivity to the periorbital region is not a general feature of injections of IL-6 into the spinal canal but that it is unique to activation of meningeal afferents or the TNC.

## Discussion

While many migraine patients report cutaneous hypersensitivity in cephalic and in one or multiple additional extracephalic sites during attack [4–6], it is less common that patients with pain states in the lower part of the body experience cephalic hypersensitivity. Little is known about how or why this occurs. Here we examined whether a similar effect occurs in rats where provoking migraine-like conditions causes body wide hypersensitivity and whether these widespread responses are absent when the same stimulus is given into locations that would cause other types of pain. We used the pro-inflammatory cytokine IL-6, which is upregulated during migraine attacks, but is a stimulus that is also implicated in pain states in other body regions [32]. IL-6 administered directly onto the dura of female rats results in facial and hindpaw hypersensitivity similarly to our previous reports in male rats [8]. In contrast, intraplantar IL-6 at the same dose only produces hindpaw responses in either females or males. Subcutaneous IL-6 onto the scalp produced no facial or hindpaw allodynia in either sex. We also show that IL-6 administered intracisternally produces facial hypersensitivity in both sexes, but only leads to whole body responses in females. Finally, when IL-6 was administered intrathecally, neither males nor females exhibit facial hypersensitivity, but females demonstrated significant decreases in hindpaw withdrawal thresholds.

Together, these data show that the only locations where administration of IL-6 leads to periorbital hypersensitivity is onto the dura or via intracisternal injection, the latter of which is capable of activating central trigeminal pathways for all innervation targets. These are also the only stimulus locations where hypersensitivity is referred to distant locations across the body. This finding suggests the presence of unique pathways through the trigeminal system that are capable of establishing widespread behavioral responses and that similar pathways do not exist within the lower spinal system. These data may help to offer additional insight into the mechanistic differences by which meningeal afferents and other sensory inputs engage central circuits to cause pain and also may aid in the understanding of how and why migraine is such a debilitating disorder.

The data presented here show a robust ability of dural stimulation with IL-6 to cause both facial and hindpaw hypersensitivity, demonstrating a role for meningeal inputs in these responses. While this may also result from the ability of dural stimuli to penetrate the CNS [33] and lead to activation of central pain targets, IL-6 is a much larger peptide than the small molecules that were observed to have these properties and is unlikely to migrate to the CNS. Additionally, our previous work showed that dural application of pH 6.0 caused body-wide hypersensitivity [20] and it is unlikely that the H^+^ concentration rises enough throughout the CNS to cause this response. This suggests that dural IL-6 likely exerts the effects observed here via activation of dural afferents near the injection site. However, intracisternal injection of IL-6 should activate these dural inputs in addition to many, if not all, other trigeminal inputs to the TNC. Thus, it is surprising that intracisternal IL-6 did not cause hindpaw hypersensitivity in males. These data may be due to the release of additional inflammatory mediators or other signaling molecules downstream of dural IL-6 that are not present following intracisternal IL-6, ultimately adding to the stimulus intensity of the former. For example, there may be release of factors such as histamine, serotonin, proteases, and other sensitizing agents from mast cells [34, 35]. Given that mast cells are more abundant in the dura mater and leptomeninges [36], the release of additional inflammatory mediators from these cells may lead to increased engagement of trigeminal pathways in response to dural IL-6. This may also explain why the facial hypersensitivity responses of males and females to intracisternal IL-6 are less robust and of shorter duration than those resulting from dural application.

We also surprisingly observed that while subcutaneous IL-6 on the scalp failed to produce hindpaw hypersensitivity, consistent with IL-6 injections into numerous other locations, it was also unable to cause hypersensitivity of the relatively nearby periorbital skin. This is despite the fact that the scalp IL-6 injection should activate or sensitize dural fibers in the periosteum that pass through the calvarial sutures [37]. Activation of these fibers has previously been demonstrated with stimuli such as KCl and inflammatory soup [38]. In contrast, capsaicin and low pH 5.0 failed to activate these afferents reliably or robustly [38]. It is thus possible that not all stimuli are able to activate these fibers and IL-6 may be among those that cause no responses. Additionally, the location of subcutaneous scalp injection may not effectively target these periosteal trigeminal afferents directly.

While many of the responses to IL-6 shown here were not sexually dimorphic, there were several notable exceptions. We show that intrathecal administration of IL-6 only leads to hindpaw responses in female rats (Fig. 6). Similarly, IL-6 applied into the cisterna magna resulted in facial hypersensitivity in both females and males, potentially via activation of pial afferents within the subarachnoid space [39, 40], but only led to hindpaw hypersensitivity in females (Fig. 5). Prior studies show that estrogen can lead to inhibition of IL-6 production and release [41, 42], but whether this leads to changes in effects of exogenous IL-6 is not clear. Recently, a potential role for spinal prolactin (PRL) has been implicated in the production of IL-6 induced hindpaw allodynia [27], as intrathecal administration of a prolactin receptor antagonist (ΔPRL) prevents responses to IL-6 in female mice. Similarly, co-injection of PRL with IL-6 increases hindpaw hypersensitivity in female mice. The increased endogenous levels of PRL in females rats compared to their male counterparts [43–45] may explain the ability of intracisternal and intrathecal IL-6 to produce hindpaw responses in females, but not in males at this dose, as it may be a more intense stimulus in females. These findings demonstrate a clear role for the trigeminal system in *producing* whole body hypersensitivity; central administration of IL-6 to the TNC leads to whole body allodynia, while central administration that does not activate the trigeminal system is incapable of eliciting a facial response.

There are several potential limitations to this study. The only stimulus tested was IL-6 and other migraine-relevant stimuli may lead to differential responses across the body; in particular, other stimuli applied to the lower body may lead to facial hypersensitivity. However, we have previously demonstrated that hindpaw injection of CGRP leads to hindpaw allodynia in female rats, but does not result in any facial hypersensitivity [19]. Next, while we did not observe widespread hypersensitivity when IL-6 was injected subcutaneously into the scalp, there may have been different findings with IL-6 injected into other trigeminal targets such as the temporomandibular joint. Stimulation of other such tissues may be more effective at referring hypersensitivity to the facial skin. Similarly, we used the gastrocnemius muscle as a representative deep tissue, but injection of IL-6 may lead to more robust and widespread referred pain from other deep tissues such as visceral organs. While colonic inflammation has been shown to induce periorbital hypersensitivity in rodents, this model required administration of dextran sodium sulfate into the drinking water for 7 days and the location of action leading to periorbital hypersensitivity is not clear [46]. Additionally, as a result of the circulation of CSF we cannot accurately state the concentration of IL-6 at the cisterna magna or intrathecally. Furthermore, given the rate and direction of CSF [47] it is possible that intracisternal IL-6 diffuses to lower targets aiding in the development of hindpaw sensitivity. Finally, IL-6 administration at this dose leads to transient behavioral responses that may better reflect signaling pathways associated with acute pain states. Testing chronic pain models in other body locations may lead to more widespread hypersensitivity, including into the facial region.

Overall, these data show the unique ability of dural and intracisternal stimulation to produce robust whole-body hypersensitivity and are consistent with the building list of studies supporting differential connections of trigeminal and dural pathways with central circuits relevant for pain. These studies raise the possibility that afferent input from the head may more effectively engage central pain and affective circuits as an enhanced protective mechanism given the importance of the brain and other sensory structures within the head. Similarly, hypotheses have been proposed that migraine evolved as a defense mechanism, and to lead to detection of potentially harmful events such as the ingestion of toxins, lack of sleep, or hunger [48]. They further support the notion that all forms of pain have evolved to contribute to the survival of an organism [49]. This idea also offers context to the common symptoms, and diagnostic criteria for migraine, photophobia and phonophobia where the increased sensitivity to lights and sounds would signal for the animals to remain in a covered location, to become less susceptible to predators [50]. In the case of rodents, they often remain immobile to heal and recover, therefore the hindpaw allodynia we observe here in response to dural and intracisternal stimulation may serve to prevent the animal from moving. This is consistent with findings that cortical spreading depression (CSD) leads to significant freezing behavior in rats [51]. Furthermore, this freezing behavior is attenuated by CGRP inhibitors [51].

## Conclusions

The findings of this study show that activation of trigeminal afferents, and in particular dural afferents, are uniquely able to generate widespread cutaneous hypersensitivity in both female and male rats. Mechanisms underlying these effects may contribute to the distinct collection of symptoms present in pain states such as migraine where sensory symptoms spread beyond the cephalic region. Better understanding of these mechanisms may lead to novel therapeutic approaches that are differentially effective for migraine compared to pain in the rest of the body.

## Data Availability

Raw data are available upon request via contacting the corresponding author: Gregory.Dussor1@utdallas.edu.

## Abbreviations

IL-6: Interleukin-6
TG: Trigeminal Ganglia
TNC: Trigeminal Nucleus Caudalis
TLR4: Toll-like Receptor 4
CGRP: Calcitonin Gene-Related Peptide
ΔPRL: Δ1–9-G129R-hPRL
PRL: Prolactin
CSD: Cortical Spreading Depression
